# Profiling of Metabolomic Changes in Plasma and Urine of Pigs Caused by Illegal Administration of Testosterone Esters

**DOI:** 10.3390/metabo10080307

**Published:** 2020-07-27

**Authors:** Kamil Stastny, Kristina Putecova, Lenka Leva, Milan Franek, Petr Dvorak, Martin Faldyna

**Affiliations:** 1 Veterinary Research Institute, Hudcova 296/70, 62100 Brno, Czech Republic; putecova@vri.cz (K.P.); leva@vri.cz (L.L.); franek@vri.cz (M.F.); faldyna@vri.cz (M.F.); 2 Faculty of Veterinary Hygiene and Ecology, University of Veterinary and Pharmaceutical Sciences Brno, Palackeho tr. 1946/1, 61242 Brno, Czech Republic; dvorakp@vfu.cz

**Keywords:** metabolomic, anabolic practices, testosterone, plasma, urine, pigs

## Abstract

The use of anabolic steroid hormones as growth promoters in feed for farm animals has been banned in the European Union since 1988 on the basis of Council Directive 96/22/EC. However, there is still ongoing monitoring and reporting of positive findings of these banned substances in EU countries. The aim of this work was to investigate the efficacy and discriminatory ability of metabolic fingerprinting after the administration of 17β-testosterone esters to pigs. Plasma and urine samples were chromatographically separated on a Hypersil Gold C18 column. High resolution mass spectrometry metabolomic fingerprints were analysed on a hybrid mass spectrometer Q-Exactive. Three independent multivariate statistical methods, namely principal component analysis, clustre analysis, and orthogonal partial least squares discriminant analysis showed significant differences between the treated and control groups of pigs even 14 days after the administration of the hormonal drug. Plasma samples were also analysed by a conventional quantitative analysis using liquid chromatography with tandem mass spectrometry and a pharmacokinetic curve was constructed based on the results. In this case, no testosterone residue was detected 14 days after the administration. The results clearly showed that a metabolomics approach can be a useful and effective tool for the detection and monitoring of banned anabolic steroids used illegally in pig fattening.

## 1. Introduction

The use of hormones as growth promoters for fattening purposes in livestock has been banned in the European Union since 1988 by Council Directive 96/22/EC. However, the banned substances are still reported as positive in the European residue monitoring plans [1]. One of these banned substances is the anabolic and androgenic steroid testosterone, which naturally occurs in animal organisms. Testosterone is endogenously secreted by Leydig cells (testes) and is able to accelerate muscle growth (anabolic effect) and improve the development of male characteristics (androgenic effect). Testosterone is then secreted into the bloodstream where it primarily (98%) binds to a specific protein beta-globulin termed sex hormone binding globulin (SHBG) and to a lesser extent to albumin. By this binding, testosterone is biologically protected from inactivation in the liver, and is subsequently transported to the target tissues via the bloodstream. A small amount of circulating testosterone is converted to estradiol, but the greater part of free testosterone is converted to 17-ketosteroids, particularly androsterone and its isomer etio-cholanolone (androsterone metabolites) [2]. In some target tissues, testosterone is reduced to 5α-dihydrotestosterone (DHT) by the cytochrome P_450_ enzyme 5α-reductase, an enzyme highly expressed in male sex organs, skin, and hair follicles. The inactivation and degradation of testosterone and its metabolites in cattle and pigs occurs mainly in the liver and, to a lesser extent, in the kidneys. These mechanisms of inactivation and degradation of testosterone occur with the participation of specific enzymes involved in the catalytic action of the partially transformed steroid molecule. Inactivation and degradation include the following: addition of two hydrogens (reduction) to a double bond or ketone group; removal of two hydrogens (oxidation) from a hydroxyl group; addition of a hydroxyl group (hydroxylation) to a carbon in the steroid molecule; and conjugation of testosterone by reaction of sulfuric acid or glucuronic acid with a hydroxyl group on the steroid molecule, forming testosterone sulphates and glucuronides, respectively. The sulfated or glucuronide conjugated form of testosterone is then excreted in the urine [3].

Testosterone (a natural steroid) is illegally administered to animals in the form of synthetic steroid esters, but these are rapidly hydrolysed to a natural steroid in vivo. For example, after oral administration of testosterone undecanoate, unchanged ester was found in athletes’ plasma for only 6 h [4]. In analytical practice, it is difficult to distinguish between metabolites of natural endogenous testosterone, which is always present in body fluids (plasma, urine), and metabolites of identical exogenous testosterone derived from hydrolysed synthetically prepared esters [5]. In human doping control, this problem is usually solved by determining the urinary ratio of 17β-testosterone/17α-testosterone levels (T/EpT ratio) or by using gas chromatography with isotopic mass spectrometry (GC-IRMS) and application of the ^13^C/^12^C isotope ratio [6]. The World Anti-Doping Agency (WADA) has established a decision limit if a T/E ratio is equal to or greater than 4, or an epitestosterone (17α-testosterone) concentration is greater than 200 ng mL^−1^ which would require a testing procedure to confirm doping [7,8]. Although important in humans, these analytical parameters have failed in animals because of differences in their metabolism [9]. In food safety practices, relatively high or low levels of 17β-testosterone and 17α-testosterone in urine are often ignored due to a lack of statistically valid reference data on naturally occurring endogenous background levels in animals. However, the EU Community Reference Laboratories (CRLs) for analytical methods recommended in the national monitoring control plans to limit concentrations for plasma (CC_α_ for confirmatory methods) to 0.5 µg L^−1^ for heifers 18 months old, 10 µg L^−1^ for bullocks six months old, and 30 µg L^−1^ for bulls 6–18 months old [10]. For other animals, no such recommendations exist for 17β-testosterone in plasma or urine.

Over time, a number of targeted analytical methods for the determination of testosterone in various biological samples (plasma, urine, muscles and hair) have been developed and described in the literature. In the 1990s, testosterone measurements were often performed by radioimmunoassay (RIA) [11] and immunoassays (ELISA) [12]. Immunological methods are fast, easy-to-perform, cheap, and have a short time to result for a large number of samples, so today they are preferably used in many laboratories primarily for screening. However, cross-reactivity and sensitivity in these assays is a common problem, so these methods are no longer good enough for the detection and quantitative determination of testosterone. 

Gas chromatography (GC) and liquid chromatography (LC) are other alternatives for the targeted analysis of testosterone and its esters. GC methods coupled with mass spectrometry (MS) are usually applied for the determination of anabolic steroid levels ranging from micrograms to nanograms in biological samples [13]. The detection of testosterone esters at 1 ng mL^−1^ in human plasma by GC/MS has been reported [14]. However, GC-MS methods require a complicated, time-consuming, and expensive step of sample derivatization for steroid analysis. In general, these derivatives are unstable and are susceptible to thermal degradation during analysis, which, in particular, significantly affects the reproducibility of the method [15]. In contrast, LC-MS is a good solution for quantitative analysis of steroids because the included sample preparation step is easy, fast, economical and requires no further derivatization step. The high performance liquid chromatography with mass spectrometry (HPLC-MS) used for the analysis of steroid esters in plasma showed greater sensitivity than GC-MS [16]. HPLC is also a commonly used separation technique for the determination of testosterone and its esters in body fluids due to its sufficient sensitivity, good resolution, robustness and short analysis time [17]. Ultra-high performance liquid chromatography (UHPLC) coupled with tandem mass spectrometry (MS/MS) or today with high-resolution (HR) mass spectrometry is another powerful approach to significantly improve peak resolution, selectivity, sensitivity and speed of the analysis [18,19,20]. It should be noted that an interesting alternative to inconclusive urine analyses (endogenous testosterone vs. synthetic testosterone) at veterinary inspection may be the analysis of intact natural steroid esters in the hair by UHPLC-MS/MS [21,22] or DESI-MS (desorption ionizing mass spectrometry) [5]. 

Recently, new synthetic xenobiotic growth promoters have been designed and new ways of application employed, such as the administration of low dose cocktails. However, metabolomics approaches to non-targeted screening for the detection of anabolic practices with natural steroid hormones might change this situation in the future [9,23]. Indeed, several scientific studies have demonstrated the efficiency of mass spectrometry with high resolution based on urinary fingerprinting to discriminate anabolised animals from control ones. Rijk et al. [24] in their work showed the use of a novel untargeted metabolomics based strategy for the measurement of the anabolic steroid DHEA (dehydroepiandrosterone) and pregnenolone in bovine urine with liquid chromatography coupled with time-of-flight mass spectrometry (LCT Premier). In the same year, Kieken et al. [25] presented a metabolomics strategy involving the characterization of global metabolomic fingerprints in urine samples of non-treated and reGH (recombinant equine growth hormone)-treated horses by LC-HRMS (LTQ-Orbitrap) as a new screening tool for growth hormone abuse in horseracing. 

Anizan et al. [26] presented in their study a metabolomics approach to 4-androstenedione (AED) detection after its administration to heifers. Using untargeted profiling by GC-MS, they identified 5α-androst-2-en-17-one in urine as a new biomarker of anabolic AED abuse. From 2011 to the present, several studies have been conducted in cattle in relation to the administration of banned substances for fattening, which have confirmed the correctness of the research focus on non-targeted analyses based on metabolomic approaches [27,28,29,30,31,32]. However, all these studies were in all cases carried out only in cattle, although in many European countries, for example, pork was consumed significantly more than beef. The only metabolomic study published so far for another animal species was conducted in 2017 in pigs to which a banned beta-agonist substance, ractopamine, was administered [33]. 

The present study aimed to investigate the efficacy of metabolomic profiling of pig plasma and urine samples by high resolution mass spectrometry (HRMS) to discriminate between the testosterone ester group and the control group. The experiment was performed in two independent groups of pigs, where individual animals were assigned to groups based on randomization. Plasma and urine samples were continuously collected at specified time intervals, prepared and subsequently measured on a high-resolution hybrid tandem mass spectrometer (QExactive). The obtained metabolomic fingerprints were processed and statistically analyzed using principal component analysis (PCA) and orthogonal partial least squares discriminant analysis (OPLS-DA) multivariate methods. Furthermore, the results of the non-targeted metabolomic analysis obtained in this way were compared with the results of the targeted determination of 17β-testosterone in the same plasma and urine samples. All pigs in the experiment were weighed at weekly intervals and the anabolic effect of testosterone was studied based on the body weight gain.

## 2. Results

### 2.1. Anabolic Effect of 17β-Testosterone (Esters)

**Figure 1:**
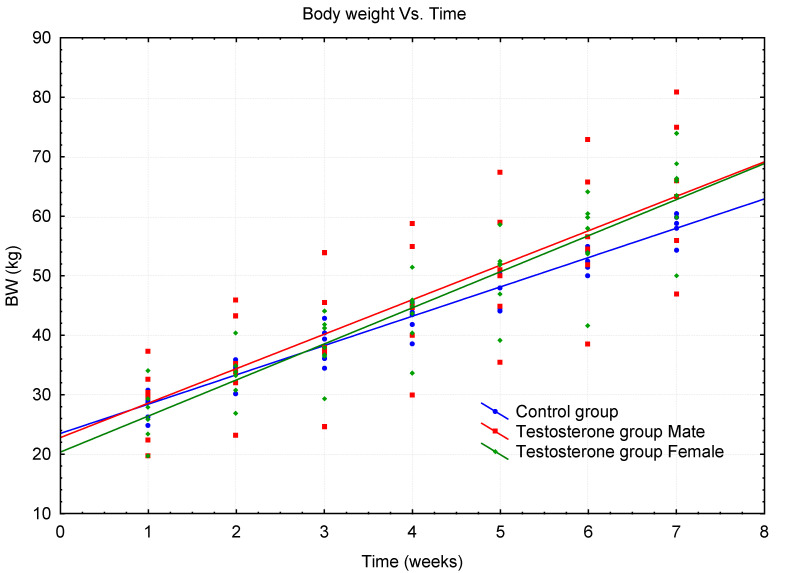
A combined diagram of two regression models for testosterone treated pigs (male and female) and the control group of pigs.

All animals from both groups were weighed at regular weekly intervals during the treatment experiment, and the body weight gain (BW) in kg is shown in Table 1 and Table 2. The anabolic effect was expressed in a graphical form of the dependence of the body weight of experimental pigs on the time interval for both treatment and control groups. Two linear regression models were used to highlight the growth trends of both groups of pigs and the statistical assessment of the anabolic effect of 17β-testosterone (Figure 1). The average weekly body weight gains were calculated from the detected BW data for each group of pigs and are shown in Supplementary Materials Table S1 and Figure S1. All animals were clinically monitored during the experiment and were in good health until slaughter at the end of the experiment. The treatment experiment was performed without any problems.

### 2.2. Targeted Analysis of 17 β-Testosterone in Plasma

#### 2.2.1. Identification of Analytes 

**Figure 2:**
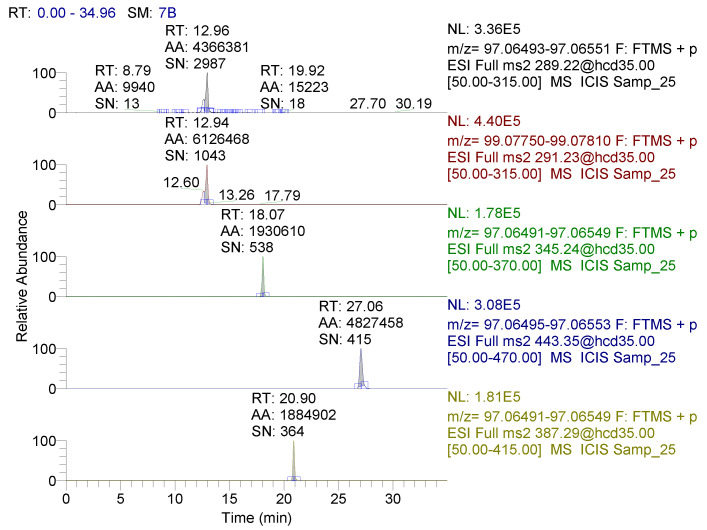
Chromatogram of the analysed plasma samples fortified with standards at 10 μg L^−1^ (ppb), RT indicates retention time, AA indicates the peak area, SN indicates signal to noise ratio, black chromatogram represents 17β-testosterone, red chromatogram represents IS 17β-testosterone-D2, green chromatogram represents 17β-testosterone propionate, blue chromatogram represents 17β-testosterone decanoate and the yellow chromatogram represents 17β-testosterone isocaproate.

Standards of 17β-testosterone, testosterone propionate, testosterone isocaproate, testosterone decanoate, and 17β-testosterone-D_2_ internal standard were always identified on the basis of the retention time (see Figure 2) obtained from the chromatogram and mass accuracy (MA) parameters calculated from mass spectra for precursor and product ions of each analyte by comparing the theoretical mass *m*/*z* with the measured experimental mass *m*/*z*. The obtained results and the calculated MA values determined by the standards are presented in Supplementary Materials Table S3 and in the diagrams showing the detected experimental mass spectra of the analyte standards, always in comparison with the theoretical mass spectra (Supplementary Materials Figures S2 and S3).

#### 2.2.2. Study Validation 

The targeted quantitative method was in-house validated complies with model-dependent performance characteristic covering specificity, selectivity, precision, repeatability, within-laboratory reproducibility, the calibration curve, detection limit (LOD), limit of quantification (LOQ), decision limit (CCα), detection capability (CCβ), and ruggedness according to the recommendation defined in Commission Decision 2002/657 / EC [34] and the reference guidelines in VICH GL49 [35].

The linearity of the quantitative method was determined for testosterone and testosterone ester analytes that were fortified into real pig plasma samples with increasing concentrations. The model samples were prepared according to the procedure described in Section 4.5. A matrix calibration curve was constructed based on the measured peak area ratios (Std. area/IS area) and the corresponding concentration levels. The parameters of the linear regression models were calculated by the least squares method (with a weight coefficient w = 1/2) based on ISO 11843: 2 [36]. Correlation coefficients (r), linear regression model parameters (y = a + bx) and critical curve limits (LOD, LOQ) were calculated and reported in Table 3. The calibration curve for 17β-testosterone, which was used to back-estimate the results of real plasma samples obtained during the experiment, was shown graphically (Supplementary Materials Figure S4). The complete standard area and internal standard area data that was used to calculate the 17β-testosterone calibration curve are presented in Supplementary Materials Table S4.

To determine the precision and repeatability (within-laboratory reproducibility) of the targeted analysis method, the standard deviation (SD) and variation coefficient (CV, %) were determined and calculated by repeated measurement of fortified plasma samples at two concentration levels. The calculated CV (*n* = 12) was less than 3.09% for a concentration level of 5 ng mL^−1^ 17β-testosterone in plasma, demonstrating the good precision and repeatability required for confirmatory residual analyses by the Commission Decision 2002/657/EC. The results of the validation study for precision, repeatability, and other calculated statistics are shown in Supplementary Materials Table S5.

#### 2.2.3. Pharmacokinetic Profile of 17β-Testosterone

**Figure 3:**
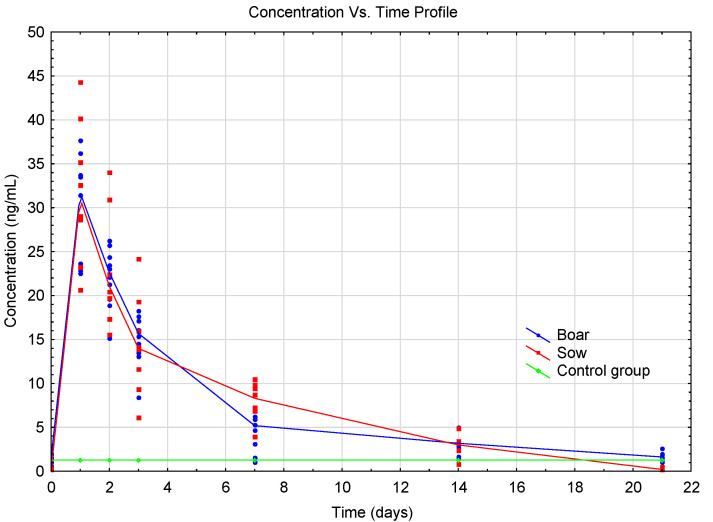
Plasma concentrations in pigs—time profile of testosterone after a single i.m. administration of 0.6 mL Sustanon 250 mg/mL inj.; the points on the curve represent the detected 17β-testosterone plasma concentrations in individual pigs.

The experiment included targeted analysis of the primary testosterone metabolite in porcine plasma after a single i.m. administration and subsequent determination of the pharmacokinetic curve. Plasma concentrations of free 17β-testosterone for individual pigs were determined based on an estimation from the matrix calibration curve (see Supplementary Materials Table S6). The resulting plasma concentrations of 17β-testosterone were used to construct a pharmacokinetic curve, and a graph of concentration versus time is shown in Figure 3. 

### 2.3. Metabolomic Study of Blood Plasma and Urine

The obtained plasma and urine samples were processed in the laboratory as described in Section 4.2. The metabolomic profiles of the individual samples on day 14 after the administration of the hormonal preparation SUSTANON were measured as described in Section 4.4. Both groups of plasma and urine metabolomic profiles were processed for a comparison in XCMS software and, alternatively, using the SIEVE company software. Both variants of data processing identified the approximately corresponding number of ions (*m*/*z*) of peaks or metabolites: 2500 ions were found in plasma and 1400 ions were found in urine. In both cases, the original number of ions in these data sets was further reduced based on a *p*-volume ≤ 0.05 for further statistical processing. The source data of sets X (n × m) after reduction each contained *n* = 21 rows (animal objects) and m = 254 columns of statistically significant identified peak areas or metabolites for plasma and m = 213 columns for urine, respectively. Datasets were transformed using two different methods, i.e., column centering [37] and probabilistic quotient normalization (PQN) [38], and a natural logarithm was applied for their scaling before the subsequent multivariate statistical analysis. The hotelling T^2^ test criterion did not identify any outlier in both data sets (Supplementary Materials Figures S5 and S6).

**Figure 4:**
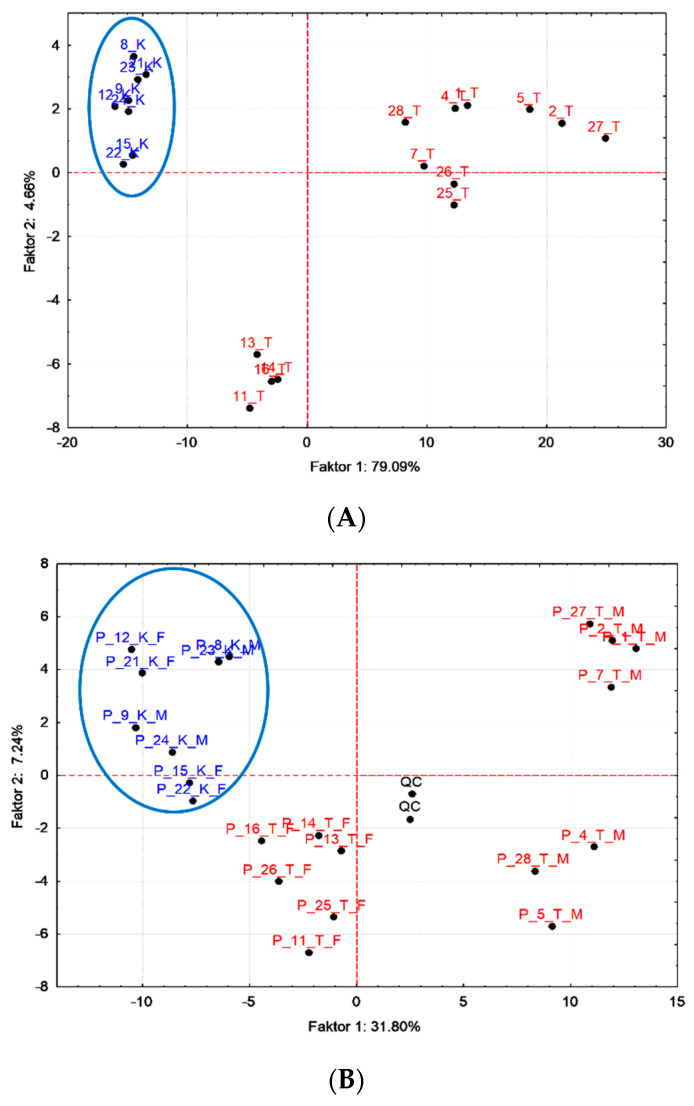
PCA Score plots for plasma (**A**) and urine (**B**) data matrix, blue ellipse, and blue point descriptions (K) represent statistically significantly different samples from the control group of pigs versus the treated (T) group of pigs; added urine labelling: M—male and F—female (Centering, STATISTICA).

**Figure 5:**
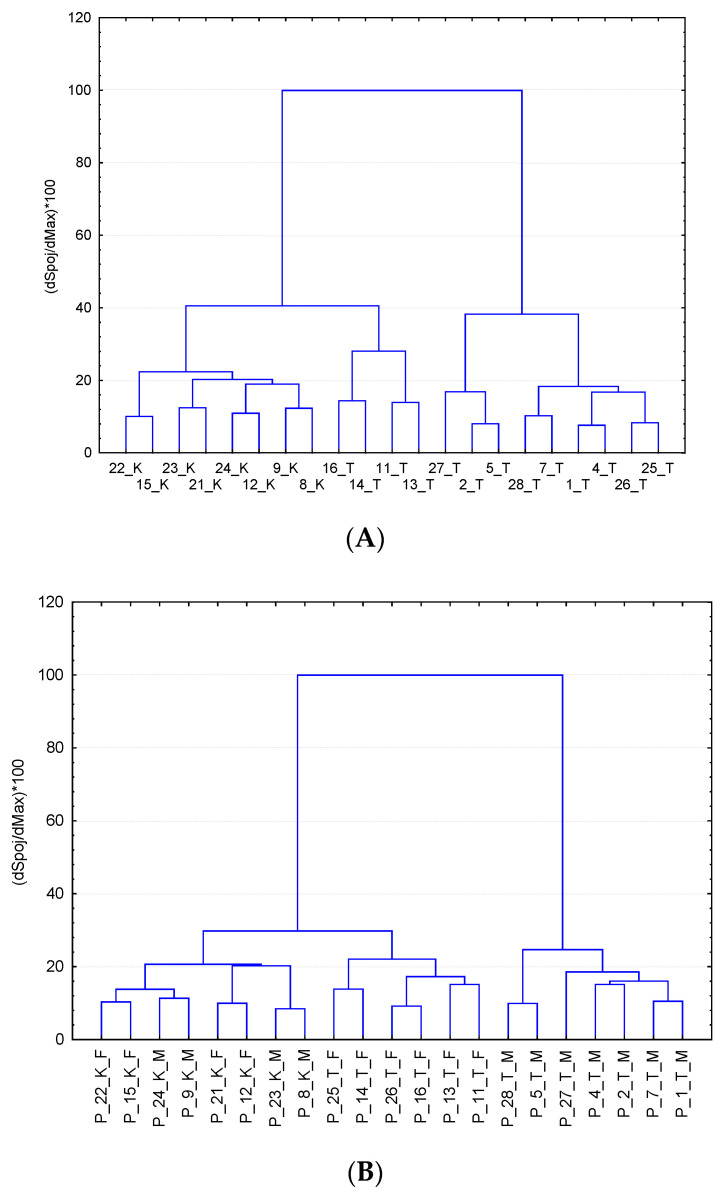
CA dendrogram of matrix data objects for plasma (**A**) and urine (**B**), labels: K—control group, T—treated group and M—male, F—female (by Euclidean distance method, STATISTICA).

**Figure 6:**
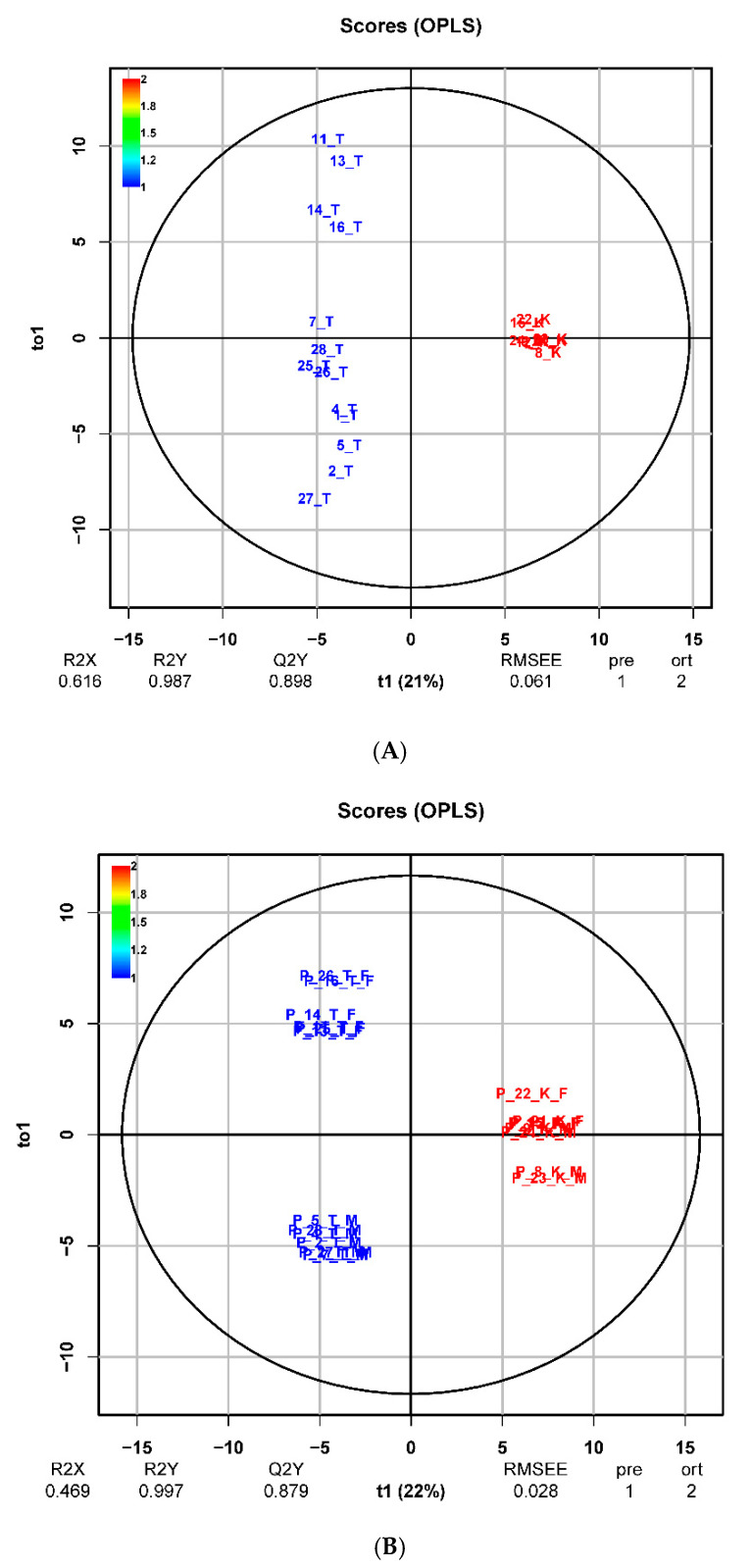
The OPLS-DA score plots for plasma (**A**) and urine (**B**) data matrix demonstrate robust discrimination between the control group of pigs marked with red colour and the group of treated pigs marked with blue colour (PQN scaling, R package). The control group indicated by red Pig number 8, 9, 12, 15, 21, 22, 23, and 24. The treated group indicated by blue Pig number 1, 2, 4, 5, 7, 11, 13, 14, 16, 25, 26, 27, and 28.

**Figure 7:**
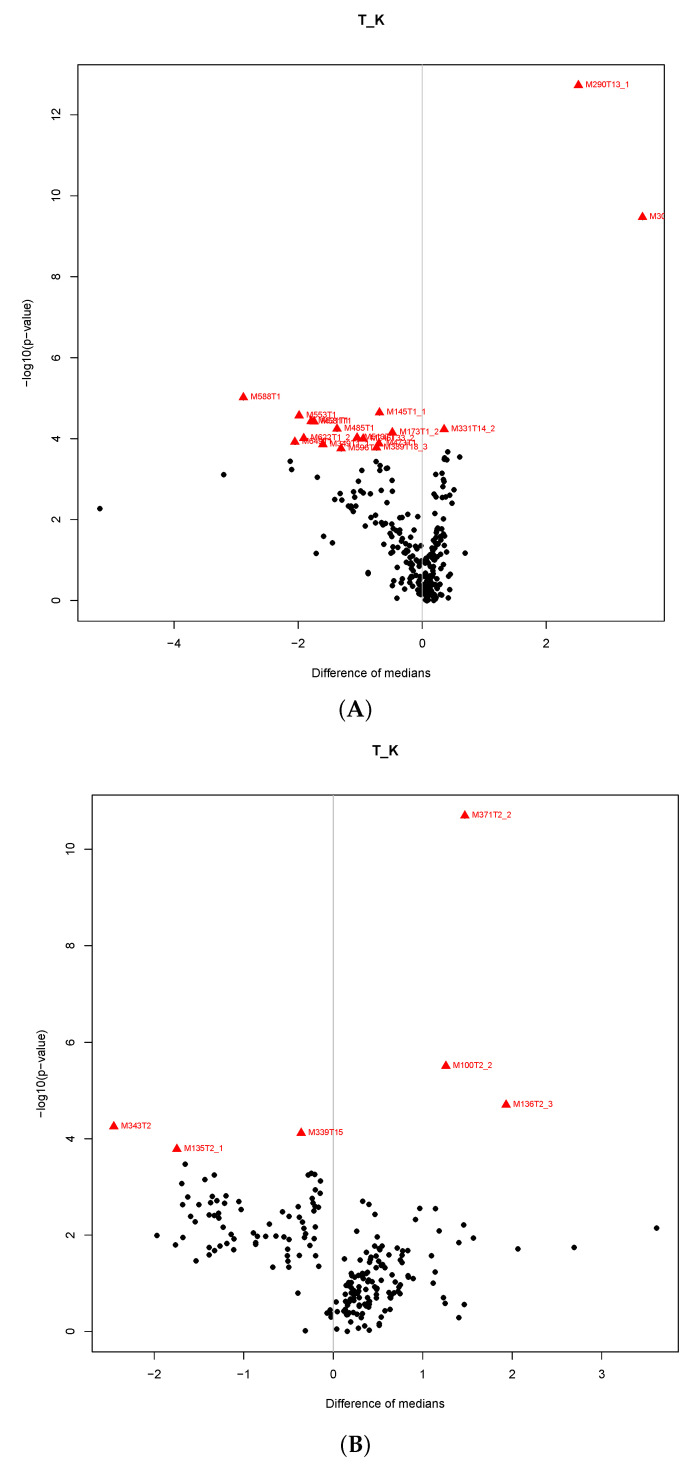
The OPLS-DA Vulcano plots for plasma (**A**) and urine (**B**) data matrix, only metabolites with the VIP scores above 2 were considered significant. A list of specific numbers of metabolites is given in Table 4.

Multidimensional statistical methods such as principal component analysis (PCA), clustering analysis (CA) and orthogonal partial squares discriminant analysis (OPLS-DA) were applied for finding relationships between metabolomics datasets of plasma and urine. PCA score plots for plasma and urine samples (Figure 4) and a dendrogram from CA (Figure 5) visibly differentiated between the control group and the treated group of pigs after 17 beta-testosterone administration. The main graphical results from the OPLS-DA analysis of data matrix of X mass spectra of plasma and urine samples versus data matrix Y for binary variables (1 = group of treated pigs, 2 = control group) were generated by the proposed statistical model and are shown in Figure 6. Furthermore, the coefficients R^2^(X) = 0.616, R^2^(Y) = 0.987 for the fit and Q^2^(Y) = 0.898 for prediction of the model (according to cross validation) were calculated by OPLS-DA analysis for plasma and the coefficients R^2^(X) = 0.469, R^2^(Y) = 0.997 and Q^2^(Y) = 0.879 for urine data. The OPLS-DA permutation tests further confirmed that the proposed statistical models are correct and robust (Supplementary Materials Figures S7 and S8). A volcano plot and variable importance in the projection (VIP) plot and S-plot from OPLS-DA were employed to determine the most discriminating metabolites between the treatment group and the control group (Figure 7). 

### 2.4. QC Samples

**Figure 8:**
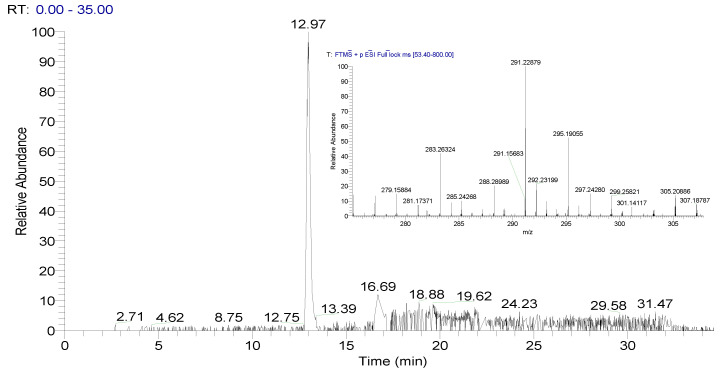
Pooled urine QC sample, control chromatogram for internal standard and corresponding mass spectrum (experimental *m*/*z* = 291.22879, ∆ppm = 0.1).

To control the quality of the metabolic profile (fingerprint) measurement, a constant amount of the internal 17β-testosterone-D2 standard was added to each plasma and urine sample (Figure 8), and pooled QC samples were included in each measured acquisition. The RT (deviation up to 10%), peak area (deviation up to 10%) and MA (∆ppm < 3) were checked in each measured metabolomic profile.

## 3. Discussion

The experimental study was carried out in pigs with the main aim of demonstrating the suitability or unsuitability of metabolomic approaches and techniques for detecting the use of banned androgenic anabolic steroids in animal feed and food of animal origin in European countries. All experimental data obtained from the performed study were statistically evaluated using interactive computer-oriented approaches and specialized statistical software with the main emphasis on the correct interpretation of results and endeavour for obtaining a more comprehensive view of the analyzed and assessed topics of our study. The results were presented primarily in a graphical form, as opposed to the mathematical evaluation of the performed statistical analyses, because they generally have a higher predictive ability for the overall evaluation of the achieved effect.

### 3.1. Anabolic Effect of 17β-Testosterone (Esters)

From the obtained experimental data of BW measurements in a weekly time interval, estimates of standard univariate statistics (mean, variance, standard deviation, median, median standard deviation and 95% confidence interval) were calculated for each time period, separately for the treated pigs and the control group (Table 1 and Table 2). The mean BW of pigs from the treated group was lower (mean = 27.7 kg, CI = [24.5; 30.9]) 1 week after 17β-testosterone administration than the mean BW of pigs from the control group (mean = 28.3 kg, CI [26.4; 30.1]). At the end of the weighing at week 7 after the administration, this situation was reversed. The mean BW of pigs from the treated group (mean = 64.4 kg, CI = [58.6; 70.2]) was 9% higher than the mean BW of pigs from the control group (mean = 58.5 kg, CI = [56.8; 60.2]) and averages differed significantly (*t*-test, *p* < 0.05).

Two linear regression models of BW growth versus time were designed to test the anabolic effect, each model especially for the group of treated pigs and the data of the control group (Figure 1). Both models were tested by regression diagnostics. Testing of the regression triplet (data + model + method) showed that the proposed linear models were significant and correct. Estimates of the regression parameters of both linear models are given in Supplementary Materials Table S2. Statistical testing of a comprehensive comparison of both models over the whole-time interval was performed. The Chow test [39] was used to test equality between sets of coefficients in two linear models, using the Fisher-Snedecor distribution with *m* and *r* degrees of freedom as statistics. The calculated statistic F_Ch_ = 13.583 was greater than the critical value of F_0.95(2.62)_ = 3.150. It could be concluded that the hypothesis H_0_ was rejected at the significance level α = 0.05 and both models were not equal. Applying the conclusion of the Chow test, it can be concluded that the results of BW growth detected in pigs in relation to the time interval can be considered different for both groups of pigs, e.g., the effect of the anabolic effect of testosterone was demonstrated. Since the initial weights of the two groups of pigs did not differ significantly, it can be stated that in this case the slope of the two linear models compared is statistically significantly different. This partial conclusion of the study is also evident from the graphical comparison of the linear curves of both groups of pigs (see Figure 1). The anabolic effect, but in this case of nandrolone (19-nor 17β-testosterone), a synthetic analogue of testosterone, has recently been similarly demonstrated in treated barrows vs. control barrows [40].

### 3.2. Targeted Determination of 17β-Testosterone in Plasma

An analytical method for the targeted determination of 17β-testosterone in pig plasma based on LC-MS/(HR)MS was developed as part of this study to estimate the pharmacokinetic curve. The quantitative method of analysis was developed and validated as a confirmatory method as required by the European Directive for residues [34]. The correct identification of targeted analytes using the mass accuracy (MA) criterion [41,42] and quantification based on a matrix calibration curve with parameter estimates for precision and repeatability were part of the validation of the confirmation method; the results are given in Section 2.2. A detailed description of the methodology used for the identification and validation of targeted analytes has been previously described by Stastny et al. [43].

All calculated MA values (Supplementary Materials Table S2) ranged from 0.1 to 1.9 (∆ppm) for the individual analytes determined, and these calculated values were lower than the allowed instrumental tolerance ≤ 3 ppm for the QExactive mass spectrometer used. The tolerance of retention times (RT) was below ± 10% in all cases. Furthermore, a very good chromatographic separation of the analytical method from the chromatogram shown in Figure 2 is evident, where the signal-to-noise ratio (SN) value shown is significantly higher than the generally recommended value (SN > 3), e.g., for 17β-testosterone, the ratio was SN = 2987:1. Correlation coefficients (r) estimated for the target analytes were ≥0.9991 (17β-testosterone), ≥0.9979 (17β-testosterone propionate) and 0.9996 (17β-testosterone decanoate) in plasma (Table 4). The obtained regression models showed good linearity. The sensitivity of the method was determined by the critical values LOD = 0.32 ng mL^−1^ and LOQ = 0.63 ng mL^−1^ for 17β-testosterone based on estimated from the matrix calibration curve model. The precision of the method as a simple repeatability, expressed as the value of the relative standard deviation (RSD), was 3.09% for 17β-testosterone to a concentration level of 5 ng mL^−1^. Based on the results obtained from the validation study, it was possible to conclude that the developed analytical method is suitable for the identification and quantification of free 17β-testosterone in pig plasma in the range of matrix calibration 0.5 to 80 ng mL^−1^.

The pharmacokinetic curve of free 17β-testosterone in pig plasma after the administration of 0.6 mL/pig of the hormonal preparation SUSTANON 250 was constructed on the basis of the results of detected concentrations in selected time intervals (Figure 3). The application dosage of the hormonal drug used was calculated on the basis of the recommended dosage for this drug in human medicine. The pharmacokinetic curves for castrated boars and sows were identical. The maximum C_max_ concentration (29.31 ng mL^−1^ ≈ 102 nmol L^−1^ for castrated pigs, 31.73 ng mL^−1^ ≈ 110 nmol L^−1^ for sows) was reached t_max_ 24 h after the administration. Plasma testosterone levels returned to the mean endogenous testosterone levels in castrated boars and sows in approximately 21 days. The PK results for pigs corresponded to the pharmacokinetic properties of i.m. administration of testosterone in men listed in the summary of product characteristics (SPC) of the hormonal drug used (C_max_ = 70 nmol L^−1^, t_max_ = 24–48 h, elimination time approximately 21 days). Although there are a number of published results on PK testosterone levels in human medicine, the authors of this study of testosterone pharmacokinetics could not compare their results with other authors because such results in animals (pigs) have not been yet published in veterinary medicine.

The determined concentrations of endogenous free 17β-testosterone in the plasma of castrated boars of the hybrid Lage White/Czech White breed (50/50) ranged from 0.55 to 2.58 ng mL^−1^ (mean = 1.56 ng mL^−1^). These determined concentrations for endogenous testosterone corresponded to the values reported in the literature: White composite breed 4.0 ng mL^−1^ [44], Yorkshire 2.5–5.1 ng mL^−1^, and Duroc 0.7–3.1 ng mL^−1^ [45]. The determined concentrations of endogenous free 17β-testosterone in sow plasma were below the limit of detection (<LOD) of the analytical method.

The individual testosterone esters contained in SUSTANON 250 hydrolyzed very rapidly in the bloodstream of pigs and were no longer detecable 24 h after application, the concentration was <LOD of the targeted quantitative method. Authors Rejtharová et al. [46] describes the methodology of targeted analysis of testosterone esters in model samples of bovine and porcine serum by LC-MS/MS. On the contrary, as the results of our study show, the targeted determination of testosterone esters in pig serum samples to directly demonstrate the illegal use of banned testosterone is not very suitable for a very short hydrolysis time after application in a real biological system.

### 3.3. Metabolomic Profiling of Changes in Plasma and Urine

The matrices of metabolomics data *X* obtained from an experiment performed in pigs and measured on a high-resolution mass spectrometer contain data of the same type *m*/*z*, i.e., they are homogeneous matrices (low molecular weight compounds, metabolites). To search for latent structure and reveal interrelationships in characters (X-variables) and mainly in objects, cases (pigs) in metric scale, two generally used multivariate statistical methods for character reduction to latent variables were used: principal component analysis (PCA) and cluster analysis (CA). To investigate the dependences between the independent matrix X of metabolomics data and the second dependent variable matrix Y (single-column matrix with binary data, treated group = 1 and control group = 2), another powerful statistical method, partial least squares projection to latent structures PLS was used in the form of differential PLS-DA analysis and in the orthogonal variant O-PLS-DA. Unsupervised PCA and supervised OPLS-DA are today the most widely used multidimensional statistical methods in metabolomics for non-targeted monitoring of changes in biochemical pathways in various biological samples, for their ability to reduce data dimensions or reduce large numbers of variables without much loss of information contained in their first few principal components (most often 2–3).

The metabolomics study was performed in a total of 21 pigs (objects), which were allocated into two groups: a treated group (*n* = 13) and a control group (*n* = 8). The problem of the first metabolomics studies in the field of food safety and illegal use of prohibited substances in livestock fattening performed and published between 2009 and 2010 was mainly the small number of experimental animals [24,25,26]. The authors of these first metabolomic studies were aware of this problem and subsequent studies carried out and published since 2011 have already been performed in an adequate number of animals (*n* > 10), for example [28,29,30,31].

The main results of the PCA method were score plots for plasma and urine data matrix (Figure 4), which showed significant discrimination of all objects (pigs) into two large clusters for both cases of biological matrices. A compact cluster of control group pigs (indicated by a blue ellipse) against two clearly separated clusters (point descriptions marked in red) of pigs from the treated group was found. In the treated group of pigs after testosterone administration, there was another incomplete discrimination according to the characteristics of the sex. However, in the case of plasma, two sows (objects 25 and 25) remained assigned to a cluster of castrated boars, which means that they correlated more with this group. The above results lead to a significant partial conclusion that the built PCA models were able to clearly differentiate the group of tested pigs from the control group.

A CA dendrogram of matrix data objects for plasma and urine (Figure 5) were constructed based on the average Euclidean distance and also showed a reliable distinction between the group of tested pigs and the control group, so CA analysis confirmed the previous partial conclusion from PCA. The following graphical outputs from the third supervised OPLS-DA method (Figure 6) scatter plots also confirmed the previous conclusions and were able to significantly differentiate the group of control pigs (marked in blue) from the group of tested pigs (descriptions marked in red). The R^2^ (X), R^2^ (Y) and Q^2^ (Y) statistics for the OPLS-DA models were calculated. Multiple correlation R^2^ and cross-validated coefficient Q^2^ for control vs. treated group R^2^ (X) = 0.616, R^2^ (Y) = 0.987 and Q^2^ (Y) = 0.898 for plasma and R^2^ (X) = 0.469, R^2^ (Y) = 0.997 and Q^2^ (Y) = 0.879 for urine, confirmed good class separation and a high predictive ability. Coefficients Q^2^ (Y) expressing the predictive abilities of the proposed model were calculated for 75% cross-validation.

**Figure 9:**
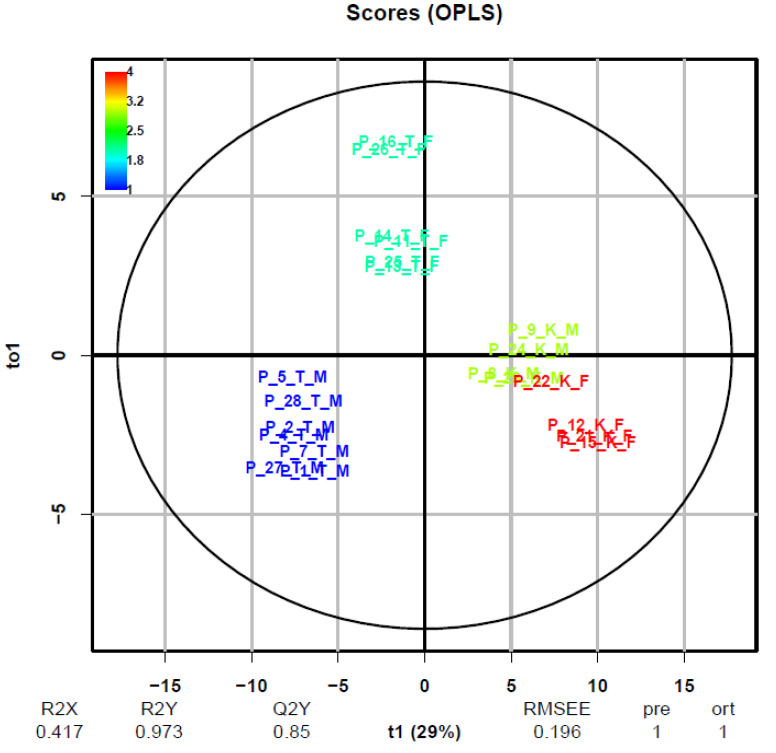
The OPLS-DA score plots for urine data matrix demonstrate discrimination between the control group of pigs and secondary discrimination by sex, objects (pigs) marked with: K—control group, T—treated group, M—male and F—female (PQN scaling, R package). A list of specific numbers of pigs is given in Table 1 and Table 2.

The group of tested pigs, analogously as in the PCA and CA models, was further divided in the plane t1 and to1 into two clusters according to their sex. Therefore, the OPLS-DA model for urine was further designed and tested, where in matrix Y (two-column matrix with binary data; treated group = 1, control group = 2 and male group = 1, female group = 2) there was a differentiation according to gender (Figure 9). Statistics R^2^ (X) = 0.417, R^2^ (Y) = 0.973, and Q^2^ (Y) = 0.85 also showed good separation and high prediction. The observed differentiation of groups of pigs by sex in all three models used after testosterone administration brings a whole new dimension to the whole issue of using metabolomics profiling to prove illegal use of banned substances. This genetic factor will have to be taken into account and further investigated in additional metabolomics studies in pigs.

All three used multivariable statistical methods, PCA, CA, and OPLS-DA, mathematically independent, were able to significantly differentiate the use of synthetic exogenous testosterone from naturally occurring (endogenous) testosterone in pigs of the same breed. This conclusion is in contrast to the findings published in the only study performed to date on ractopamine (a group of banned β-agonists) in 2017 [33]. Here, the authors of this study state that no significant difference between the samples from the control group and the ractopamine treated group was observed in the PCA analysis when all features were used. The use of metabolomics approaches and techniques also seems to be very promising from the point of view of the time of detection of banned anabolic steroids. Synthetic exogenous 17β-testosterone was demonstrably detected in plasma and urine in the treated group of pigs 14 days after administration. A similar conclusion was reached by the authors of a metabolomics study performed in cattle with β-agonists [31], when urine samples taken on days 27 and 48 after the administration could no longer be distinguished from the control group using PCA and OPLS-DA statistical models.

Supervised OPLS-DA models for urine and plasma, as one of the important practical results of this work, will be used for further testing of real samples to verify their predictive abilities. The models will be supplemented with other banned anabolic steroids and, especially, with increased numbers of test (training) data. Subsequently, the models will be verified by screening real plasma and urine samples taken as part of the monitoring of foreign substances in the Czech Republic. This is in line with the findings of other metabolomics studies [28,30,31,32], which also suggest increasing the number of samples used in statistical models and testing the proposed models on real samples obtained from national monitoring of contaminants in other EU countries (e.g., France, The Netherlands, Spain).

### 3.4. Metabolomic Profiling for Identification of Biomarkers

In the sequence of metabolomics multivariate statistical analysis, the last and often the most time-consuming step is to identify the most discriminating metabolites which are essential from the point of view of elucidating metabolomics pathways. A volcano plot with variable importance in projection plot (VIP) and S-plot from OPLS-DA were used to determine the most discriminating metabolites between the treatment group and control group (Figure 7 and Supplementary Materials Figure S9). The most discriminating compounds found were compared with the METLIN database, and their list and characteristics are shown in Table 4. Some very discriminating compounds have been identified with known human testostrone metabolites and confirmed against standards based on RT and MA criteria, such as M290T13_1 which corresponds to 5α-dihydrotestosterone. Nevertheless, further work needs to be done to identify the compounds and gain detailed explanation of their chemical structure.

## 4. Materials and Methods

### 4.1. Animal Experiment and Urine/Plasma Sampling

The animal experiments were performed at the Veterinary Research Institute in Brno, Czech Republic. Twenty clinically healthy 90-day-old male and female pigs (approximately 28 kg body weight) were randomly assigned to test (13 animals) and control (8 animals) groups. Animals from the test group were treated with an i.m. injection (0.6 mL/pig) of the hormonal preparation (30 mg mL^−1^ 17β-testosterone propionate, 60 mg mL^−1^ 17β-testosterone phenylpropionate, 60 mg mL^−1^ 17β-testosterone isocaproate, 100 mg mL^−1^ 17β-testosterone decanoate; Sustanon 250, N.V. Organon, CZ Reg.56/357/91-C). Experimental animals were grower pigs (hybrids of Large White × Landrace (sow) × Duroc (boar)) which were fed twice a day with a standard commercial diet according to the weight category. Pigs were housed in two separate pens (one pen/treatment and one pen/control) of 2.80 × 2.00 m. 

The animals were injected on day 12 after the start of the experiment and were euthanized on day 90 of the experiment. Urine samples were collected from both groups 14, 28, 42 and 90 days after treatment and all samples were kept frozen until analysis at −20 °C. Plasma samples were collected from day 1, 2, 3, 4, 5, 7, 14, and 28 after treatment to day 90. After blood clotting and 10 min of centrifugation at 6000× *g* of the samples, serum was removed and kept frozen until analysis at −20 °C. All pigs were weighted every week within the experiment. All pigs were slaughtered at a body weight of 90–110 kg and the treated animal carcasses were destroyed. The study was performed in compliance with Act No. 246/1992 Coll. of the Czech National Council for the protection of animals against cruelty and with the agreement of the Branch Commission for Animal Welfare of the Ministry of Agriculture of the Czech Republic (permission no. MZe 17214).

### 4.2. Reagents and Materials

Reference analyte standards (17β-testosterone, 17β-testosterone-D2 as isotopically labeled internal standards) were purchased from Sigma-Aldrich, Prague, Czech Republic. The standards were dissolved in methanol, diluted to a low concentration (mg mL^−1^) and used as working solutions. The organic solvents used were obtained from Merck (Darmstadt, Germany) and were in the SupraSolv^®^ class. Used water prepared in an ultrapure water system of Golgman’s water (Prague, Czech Republic). Centrifugal membrane filters Vivacon 500, cut off at 10 kDa, were obtained from Sartorius, Prague, Czech Republic.

### 4.3. Sample Preparation

#### 4.3.1. Plasma Samples for Targeted Analysis

Each defrosted plasma sample (500 μL) was transferred to a 15 mL centrifuge tube. After adding a methanol solution of 50 μL of 17β-testosterone-D2 internal standard and 5 mL of ethyl acetate, the samples were shaken vigorously for 3 min on a vortex and then centrifuged at 4000× *g* for 10 min. The supernatant (ca. 4 mL) was subsequently transferred to evaporator tubes. The samples were evaporated to dryness at 25 °C using a gentle stream of nitrogen (99.99% N_2_). The samples were reconstituted with 200 μL in mobile phase solution (methanol: water, 70:30, *v*/*v*) and filtered through a 0.45 μm (Hydrophilic PTFE) membrane centrifuge filter. The resulting samples were transferred to the insert in chromatography vials (250 µL). Then, 10 μL samples were injected directly the LC-MS/MS system.

#### 4.3.2. Samples for Metabolomics Profiling 

Plasma samples were defrosted at room temperature, homogenized and normalized by the creatinine concentration (Chemistry Analyzer BS-200, MINDRAY, Nanshen, China). Subsequently, 200 μL of each sample was filtered through centrifugal devices (Vivacon 500, cut-off at 10 kDa, 14,000× *g*, 4 °C, 30 min) to remove high molecular weight proteins. Filtrates (120 μL) were mixed with 30 μL of internal standard (testosterone-D_2_ in methanol at the concentration of 1 ng mL^−1^). After thorough shaking, 5 μL of each sample was injected into the chromatographic system.

Urine samples were defrosted at room temperature and normalized by specific density adjustment [47]. The specific density of the samples was measured using a refractometer (digital refractometer 30GS, Mettler-Toledo, Prague, Czech Republic) and, if necessary, adjusted to approximately 1.010–1.030 kg m^−3^ by dilution with deionized water. The urine samples (500 μL) were then centrifuged through a centrifugal membrane filter (Vivacon 500, cut-off 10 kDa) at 14,000× *g*, 4 °C for 30 min to remove high molecular weight proteins. To the sample filtrate (ca. 450 μL), 50 μL of 17β-testosterone-D2 internal standard was added. The urine samples were transferred to chromatographic vials and subsequently analysed.

QC samples for quality control of metabolomics profiling were prepared by the pool method so that they have the same or very similar (bio)-chemical varieties in the same range as individual samples of the study. The QC sample was prepared as a pool of all individual plasma or urine samples that were included in the study. Each QC sample was generated by mixing 20 μL of the filtrate (cut-off) of each individual sample and was analysed at the same time as the study samples as part of the overall measurement sequence.

### 4.4. Targeted Quantitative Analytical Method

#### 4.4.1. LC Condition

Plasma samples were injected directly into a Thermo Fisher Scientific, (Waltham, MA, USA) LC system Accela 1200 equipped with an autosampler with a temperature controlled tray and column. Chromatographic separation was performed on Waters C18 XTerra MS analytical columns (150 × 2.1 mm, size 3.5 μm) with a Waters C18 XTerra MS guard column (10 × 2.1 mm, size 3.5 μm). The column and autosampler tray temperatures were set at 35 °C. The mobile phase consists of 0.1% formic acid in water: methanol (95:5, *v*/*v*) A and 0.1% formic acid in water: methanol (5:95, *v*/*v*) B, the flow rate was constant 300 µLmin^−1^. Gradient elution of 0–2 min with mobile phase 95% A and 5% B was started, 2.1–20 min linear gradient from 5% to 90% B, 20.1–25 min 10% A and 90% B, 25.1–30 min linear gradient from 5% to 95% A and 30.1–35 min 95% A and 5% B. The runtime of the method was 35 min.

#### 4.4.2. MS/MS Parameters

The tandem hybrid mass spectrometer Q Exactive (Thermo Fisher Scientific, (Massachusetts, USA) equipped with a heated electrospray ionisation probe measured in a positive mode (H-ESI+). For targeted quantification analysis, the mass spectrometer worked in the parallel reaction monitoring mode PRM (corresponding to the selected reaction monitoring mode SRM) with high resolution RP = 17,500 (FWHM) at 200 *m*/*z*. Before the start of each acquisition series, the mass spectrometer was externally calibrated to the mass accuracy with the positive ion calibration solution and the negative ion calibration solution (both Thermo Fisher Scientific). The “lock-mass” calibration was set to the molecular mass of [M+Na]^+^ = 64.01577 g mol^−1^ and [M_2_+H]^+^ = 83.06037 g mol^−1^ for the acetonitrile ion, and was run continuously during the acquisition. Instrument and collision cell (HCD) parameters were optimised by direct syringe infusion of working solutions of 50 ng mL^−1^ of each targeted compound with a 5 µL min^−1^ flow-rate. The mass spectrometer setting was as follows: sheath gas flow rate 30 (unit), aux gas flow rate 5 (unit), spray voltage 4.0 kV, capillary temperature 320 °C, heater temperature 220 °C, S-lens RF level 50, AGC target of 5 × 10^6^, collision energy 35 eV, and a maximum injection time of 100 ms.

The precursor ions and the four most intense product ions for each analyte were measured for quantification and identification (confirmation), respectively. The whole LC-(HR) MS system was controlled and the acquired data were stored and processed using Xcalibur 3.1 software, and then evaluated using Mass Frontier v. 7.0 for the identification.

#### 4.4.3. Method Validation

The targeted quantitative method for the determination of 17β-testosterone and its esters in plasma has been validated to the extent required by European Directive 657/2002/EC [34] used for the determination of residues of foreign substances in biological matrices and according to the VICH GL49 [35] reference guide for validation method. To determine the validation characteristics of the matrix calibration curve, critical values detection limit (LOD), limit of quantification (LOQ), decision limit (CC_α_), detection capability (CC_β_) and calibration range 0.5–80 ng mL^−1^ was used for 17β-testosterone and its esters. Samples of pig plasma (blank) were supplemented with the standards of 17β-testosterone and its esters at concentration corresponding to 0.5, 1, 5, 10, 20, 40, and 80 ng mL^−1^. The concentrations of the internal standards were constantly 10 ng mL^−1^. For each concentration level, two model samples were prepared and each sample was measured two times. Linear regression was carried out by plotting the peak area ratios of the analyte against the internal standard (dependent variable Y) versus the analyte concentration (independent variable X). To evaluate the precision of the method, repeatability and within-laboratory reproducibility, standard deviation (SD), and the coefficient of variation (CV, %) were determined. Six model samples of pig plasma (*n* = 6) were prepared at concentrations of 5 and 80 ng mL^−1^ for all standards and measurements which were repeated two times for each sample on three different days (3 × 6, *n* = 18).

### 4.5. Metabolomic Profiling

#### 4.5.1. LC Separation

Plasma and urine samples prepared for non-targeted analysis (fingerprinting) were injected directly into the Accela 1200 LC system with a mass spectrometer. Chromatographic separation was performed on a C18 Hypersil GOLD (50 × 2.1 mm, 1.9 μm size) analytical column equipped with a C18 Hypersil GOLD (10 × 2.1 mm, 1.9 μm size) guard column, both from Thermo Fisher Scientific. The temperatures of the column and the autosampler tray were set at 35 °C and 20 °C, respectively. The flow rate through the column was constantly set at 200 μL min^−1^. The injection volume of the sample was 5 μL. The mobile phase consists of 0.1% formic acid in water: methanol (95:5, *v*/*v*) A and 0.1% formic acid in water: methanol (5:95, *v*/*v*) B, the flow rate was constant 300 µL min^−1^. Gradient elution of 0–2 min with mobile phase 95% A and 5% B was started, 2.1–20 min linear gradient from 5% to 90% B, 20.1–25 min 10% A and 90% B, 25.1–30 min linear gradient from 5% to 95% A and 30.1–35 min 95% A and 5% B. The runtime of the method was 35 min.

#### 4.5.2. Non-Targeted Mass Spectrometry

For non-targeted metabolomic analysis, the tandem hybrid mass spectrometer Q Exactive worked in the positive full scan mode with resolving power (PR) = 70,000 (FWHM) at 200 *m*/*z* in the range 50 to 750 *m*/*z* and in the centroid mode. Before the start of each acquisition series, the mass spectrometer was externally calibrated to the mass accuracy with a positive ion calibration solution and a negative ion calibration solution (both Thermo Fisher Scientific (Massachusetts, USA). The “lock-mass” calibration was set to the molecular mass of [M+Na]^+^ = 64.01577 g mol^−1^ and [M_2_+H]^+^ = 83.06037 g mol^−1^ for the acetonitrile ion, and was run continuously during the acquisition. The mass spectrometer setting was as follows: sheath gas flow rate 30 (unit), aux gas flow rate 5 (unit), spray voltage 4.5 kV, capillary temperature 320 °C, heater temperature 180 °C, S-lens RF level 50, AGC target of 6 × 10^6^ and a maximum injection time of 200 ms. The whole LC-(HR)MS system was controlled and the obtained data were stored and processed using Xcalibur 3.1 software.

### 4.6. Data Processing

The generated metabolomics profiling data sets were processed by the control software of the Xcalibur^®^ mass spectrometer and saved in a specific data format (*.raw). The first step was to convert data from Excalibur-specific raw files to open format files (*.mzXML) using MS Convertor software (ProteoWizard) [48]. Subsequently, metabolomics data were processed using the XCMS Online web version platform [49]. All results and images for processing were downloaded as zip files for offline analysis, including putative METLIN identities for each metabolite.

### 4.7. Statistical Data Analysis

Univariate and multivariate statistical analysis was performed in an interactive manner using statistical software Statistica (Version 13.3, TIBCO Software, Palo Alto, CA, USA) and R-statistic software in the Metabol package [50]. The data for processing was exported in the form of data matrix X (n × m) from an Excel file (output from data processing) to individual statistical programs. Before the application of multivariate statistical analysis, exploratory data analysis was conducted [51]. This included the assessment of primarily found outlier objects (or features thereof), assuming linear relationship and verifying the data provided (normality, non-correlation, homogeneity). Subsequently, the data matrices were standardized by two different methods. In the first case, the data were transformed by mean centering [37], and in the second case, they were transformed by probabilistic quotient normalization PQN [38,52] and a natural logarithm was applied for their scaling before subsequent statistical analysis. Multidimensional statistical methods, such as principal component analysis (PCA), cluster analysis (CA), and orthogonal partial least squares discriminant analysis (OPLS-DA), were used to statistically evaluate data obtained from non-targeted metabolomics analyses.

## 5. Conclusions

The present study confirmed the ability of metabolomics approaches and techniques to significantly differentiate pigs administered an androgenic anabolic steroid which is on the list of banned substances (17β-testosterone) in EU countries from control pigs. Using metabolomics profiling for plasma and urine samples, it was possible to differentiate the used synthetic exogenous testosterone from naturally occurring (endogenous) testosterone based on the results from three statistical models PCA, CA and OPLS-DA. The metabolomics workflow was designed with widely used multivariate statistical methods, analytical techniques, and equipment so that, based on the results of our study, it will be possible to develop validated methodologies for routine screening to prove the illegal use of prohibited substances in pig fattening. Furthermore, the anabolic effect of testosterone in pigs was demonstrated by comparing BW gains during the fattening period, and the targeted analysis of plasma testosterone levels provided data for the pharmacokinetic curves.

## Figures and Tables

**Table 1:** Body weight of the individual pigs treated with a hormone preparation containing a testosterone ester combination.

N_i_	Ear Number	Sex	BW (kg)/Week						
			1	2	3	4	5	6	7
1	1	♂	28.1	35.0	41.3	45.0	52.1	58.0	69.0
2	2	♂	29.4	33.4	36.9	43.8	52.1	53.8	63.6
3	4	♂	34.1	40.5	44.2	51.6	58.8	64.2	74.0
4	5	♂	26.0	30.8	36.6	45.5	52.0	60.5	66.5
5	7	♂	23.5	27.0	29.5	33.7	39.2	41.8	50.2
6	11	♀	19.9	23.2	24.7	30.0	35.6	38.6	47.0
7	13	♀	29.6	33.9	37.3	45.0	50.2	54.6	63.5
8	14	♀	37.4	43.4	45.5	55.0	59.2	65.8	75.0
9	16	♀	30.4	35.3	37.4	44.8	51.2	56.7	66.0
10	25	♀	32.7	32.0	38.0	40.0	45.0	52.0	56.0
11	26	♀	22.5	46.0	54.0	59.0	67.5	73.0	81.0
12	27	♂	19.9	34.0	38.0	40.5	47.0	54.0	60.0
13	28	♂	26.7	36.5	42.0	46.0	52.5	60.0	66.0
Female									
Average			26.8	33.9	38.4	43.7	50.5	56.1	64.2
CV			20.1	18.2	23.3	30.5	36.7	53.0	57.2
SD			4.5	4.3	4.8	5.5	6.1	7.3	7.5
CI (95%) lower			22.7	30.0	33.9	38.6	44.9	49.3	57.2
CI (95%) upper			31.0	37.8	42.8	48.8	56.1	62.8	71.2
Median			26.7	34.0	38.0	45.0	52.1	58.0	66.0
SD_m_			3.6	3.4	3.7	4.6	5.0	5.7	6.1
Male									
Average			28.8	35.6	39.5	45.6	51.5	56.8	64.7
CV			42.3	67.7	95.4	108.7	122.4	140.5	152.8
SD			6.5	8.2	9.8	10.4	11.1	11.9	12.4
CI (95%) lower			21.9	27.0	29.2	34.7	39.8	44.3	51.8
CI (95%) upper			35.6	44.3	49.7	56.6	63.1	69.2	77.7
Median			30	34.6	37.7	44.9	50.7	55.7	64.8
SD_m_			4.5	5.8	7.5	7.4	8.1	8.8	8.7

**Table 2:** Body weight of the individual pigs in the control group.

N_i_	Ear Number	Sex	BW (kg)/Week						
			1	2	3	4	5	6	7
1	8	♂	26.1	31.1	36.0	43.0	49.0	51.9	57.0
2	9	♂	26.3	30.2	34.6	38.6	44.2	48.1	54.5
3	12	♀	28.8	33.9	37.5	43.5	50.4	52.6	58.9
4	15	♀	29.2	33.8	36.1	43.5	50.0	54.2	60.5
5	21	♀	29.4	33.1	38.0	42.5	48.0	52.1	58.6
6	22	♀	30.4	36.0	43.0	45.5	51.0	55.0	60.0
7	23	♂	30.9	34.5	40.5	44.0	48.0	52.5	60.5
8	24	♂	24.9	35.0	39.5	42.0	44.5	51.5	58.1
Average			28.3	33.5	38.2	42.8	48.1	52.2	58.5
CV			4.8	3.8	7.5	4.0	6.6	4.2	4.1
SD			2.2	1.9	2.7	2.0	2.6	2.1	2.0
CI (95%) lower			26.4	31.8	35.9	41.1	46.0	50.5	56.8
CI (95%) upper			30.1	35.1	40.4	44.5	50.3	54.0	60.2
Median			29.0	33.9	37.8	43.3	48.5	52.3	58.8
SD_m_			1.5	1.5	2.1	1.8	1.7	1.8	1.5

**Table 3:** Regression parameters of matrix calibration curves in the concentration range 0 to 80 ng mL^−1^.

Analyte	Intercept (*a*)	Slope (*b*)	SD of the Slope	Correlation Coefficient *r*	LOD (ng mL^−1^)	LOQ (ng mL^−1^)
17β-testosterone	0.0518	0.0690	0.00096	0.9982	0.32	0.63
17β-testosterone propionate	0.0291	0.0482	0.00086	0.9991	0.19	0.52
17β-testosterone decanoate	0.0115	0.0713	0.00089	0.9979	0.21	0.54
17β-testosterone isocaproate	0.0248	0.0448	0.000073	0.9996	0.17	0.43

Note: LOD and LOQ were estimated according to IUPAC (Direct Signal Method) methodology.

**Table 4:** An overview of the most discriminating compounds (metabolites) found based on Vulcano-plots, VIP’s and S-plots from OPLS-DA; x—statisticaly significant.

No.	Name	*p*-Value	*m*/*z*	RT	Putative Metabolite	Vulcano	VIP	S-Plot	Name	*p*-Value	*m*/*z*	RT	Putative Metabolite	Vulcano	VIP	S-Plot
	Plasma samples								Urine samples							
1	M145T1_1	1.26 × 10^−8^	144.96248	0.75		x		x	M127T5	3.60 × 10^−3^	127.01611	4.93			x	x
2	M288T13_3	8.58 × 10^−5^	288.34193	13.10			x	x	M135T2_1	7.30 × 10^−3^	135.00340	1.65		x	x	
3	M290T13_1	1.95 × 10^−5^	290.21876	12.96	C_19_H_30_O_2_, DHT	x	x	x	M136T2_3	2.70 × 10^−3^	136.07603	1.65		x	x	x
4	M303T3_1	4.81 × 10^−5^	303.12451	3.41	C_17_H_19_O_5_, myco-phenolic acide	x	x	x	M228T12	4.63 × 10^−2^	228.19606	11.98			x	x
5	M349T1_1	1.88 × 10^−9^	348.92403	0.74		x	x		M228T6	3.51 × 10^−2^	228.15024	5.57			x	x
6	M417T1	5.91 × 10^−8^	416.91155	0.74			x	x	M271T20	2.42 × 10^−2^	271.20530	20.10	C_19_H_28_O_2_-H_2_O, Androstane-dione, DHA, DHEA	x	x	
7	M485T1	8.63 × 10^−11^	484.89886	0.74		x	x	x	M339T15	0.90 × 10^−4^	339.17783	14.66				x
8	M553T1	2.50 × 10^−8^	552.88600	0.75		x	x	x	M343T2	4.03 × 10^−2^	342.85174	1.68		x	x	x
9	M581T1	1.25 × 10^−9^	580.88112	0.74		x	x	x	M371T2_2	1.80 × 10^−3^	371.22826	1.66		x	x	x
10	M588T1	3.50 × 10^−9^	587.88214	0.74		x	x	x	M399T3	3.06 × 10^−2^	399.11460	2.55			x	
11	M596T1	5.38 × 10^−6^	595.86801	0.75		x	x	x	M441T14	3.73 × 10^−2^	441.21007	13.68			x	
12	M601T1_2	6.98 × 10^−5^	601.38312	0.75			x	x	M476T2	2.27 × 10^−2^	476.30732	2.18			x	
13	M621T1	5.28 × 10^−7^	620.87317	0.74		x	x	x	M520T3	2.65 × 10^−2^	520.33401	2.59			x	
14	M622T1_2	5.39 × 10^−8^	621.87566	0.75		x	x	x	M667T31	1.43 × 10^−2^	666.61850	30.81			x	x
15	M632T30	6.82 × 10^−5^	632.17150	30.35			x	x								
16	M633T30	3.76 × 10^−6^	633.16927	30.36			x	x								
17	M649T1	8.55 × 10^−9^	648.86805	0.74		x	x									

Note: DHA—Dehydroandrosterone, DHEA—Dehydroepiandrosterone, DHT—5α-dihydrotestosterone.

## Supplementary Materials

The following are available online at https://www.mdpi.com/2218-1989/10/8/307/s1, Figure S1: Graph of average weekly weight gains in kg, Figure S2: The measured experimental MS1 of the 17β-testosterone standards (up), comparison with the theoretical MS1 (down), Figure S3: The measured experimental MS1 of the 17β-testosterone-D2 standards (up), comparison with the theoretical MS1 (down), Figure S4: Calibration curve of 17β-testosterone in plasma, Figure S5: Hotelling plot for identification of outliers objects in data source matrix X with calculated critical value T2 for plasma, (K—control grup vs. T—treated grup), Figure S6: Hotelling plot for identification of outliers objects in data source matrix X with calculated critical value T2 for urine, Figure S7: The PLS-DA score plots for plasma (A) and urine (B) data matrix demonstrates robust discrimination between the control group of pigs marked with blue color and the group of teated pigs marked with red color (Centering, Statistica), Figure S8: The OPLS-DA permutation tests further confirmed that the proposed statistical models are correct and robust; A—plasma, B—urine, Figure S9: Variable importance in projection (VIP) and S-plots from OPLS-DA were used to determine the most discriminating metabolites between treatments and controls, A—plasma and B—urine, Table S1: The average weekly body weight gains, Table S2: The regression parameters of both linear models of BW growth versus time, Table S3: Identification and confirmation of 17β-testosterone and testosterone esters by mass accuracy (MA) for MS1, MS2 data, Table S4: Linearity of 17β-testosterone in plasma, Table S5: Precision, repeatability and within-laboratory reproducibility, Table S6: Pharmacokinetic of testosterone in plasma.
