# Supplementary material for: Profiling of Metabolomic Changes in Plasma and Urine of Pigs Caused by Illegal Administration of Testosterone Esters
Source: Metabolites. 2020 Jul 27;10(8):307. doi: 10.3390/metabo10080307 (PMC7463996; doi:10.3390/metabo10080307)

Supplemental file

Profiling of metabolomic changes in plasma and urine of pigs caused by illegal administration of testosterone esters

Kamil Stastny[1,*], Kristina Putecova[1], Lenka Leva[1], Milan Franek[1], Petr Dvorak[2] and Martin Faldyna[1]

[1] Veterinary Research Institute, Hudcova 70, CZ-62100, Brno, Czech Republic

[2] The University of Veterinary and Pharmaceutical Sciences Brno, Palackeho tr. 1946/1, CZ-612 42, Brno, Czech republic

*****Corresponding author:
E-mail address – stastny@vri.cz (Kamil Stastny)

CONTENTS page

Anabolic effect of 17ß-testosterone (esters)

Table S1. The average weekly body weight gains 3

Figure S1. Graph of average weekly weight gains in kg 3

Table S2. The regression parameters of both linear models of BW growth versus time 3

Identification of analytes (confirmation)

Table S3. Identification and confirmation of 17β-testosterone and testosterone esters by mass accuracy (MA) for MS^1, MS^2 data 4

Figure S2. The measured experimental MS^1 of the 17β-testosterone standards (up), comparison with the theoretical MS^1 (down) 4

Figure S3. The measured experimental MS^1 of the 17β-testosterone-D2 standards (up), comparison with the theoretical MS^1 (down) 4

Study validation

Table S4. Linearity of 17β-testosterone in plasma 5

Figure S4. Calibration curve of 17β-testosterone in plasma 5

Table S5. Precision, repeatability and within-laboratory reproducibility 6

Pharmacokinetic profile of 17β-testosterone

Table S6. Pharmacokinetic of testosterone in plasma 6

Metabolomic study of blood plasma and urine

Figure S5. Hotelling plot for identification of outliers objects in data source matrix X with calculated critical value T2 for plasma, (K – control grup vs. T – treated grup) 7

Figure S6. Hotelling plot for identification of outliers objects in data source matrix X with calculated critical value T2 for urine 7

Figure S7. The PLS-DA score plots for plasma (A) and urine (B) data matrix demonstrates robust discrimination between the control group of pigs marked with blue color and the group of teated pigs marked with red color (Centering, Statistica)... 8

Figure S8. The OPLS-DA permutation tests further confirmed that the proposed statistical models are correct and robust; A – plasma, B – urine 8

Figure S9. Variable importance in projection (VIP) and S-plots from OPLS-DA were used to determine the most discriminating metabolites between treatments and controls, A – plasma and B – urine 9

Table S1. The average weekly body weight gains

Group	2. week	3. week	4. week	5. week	6. week	7. week
Treated boar	5.07	4.50	5.94	7.59	6.02	7.53
Treated sow	5.05	4.33	6.32	5.86	5.79	7.89
Control pigs	4.53	4.63	4.32	5.17	3.96	6.78

Figure S1. Graph of average weekly weight gains in kg.

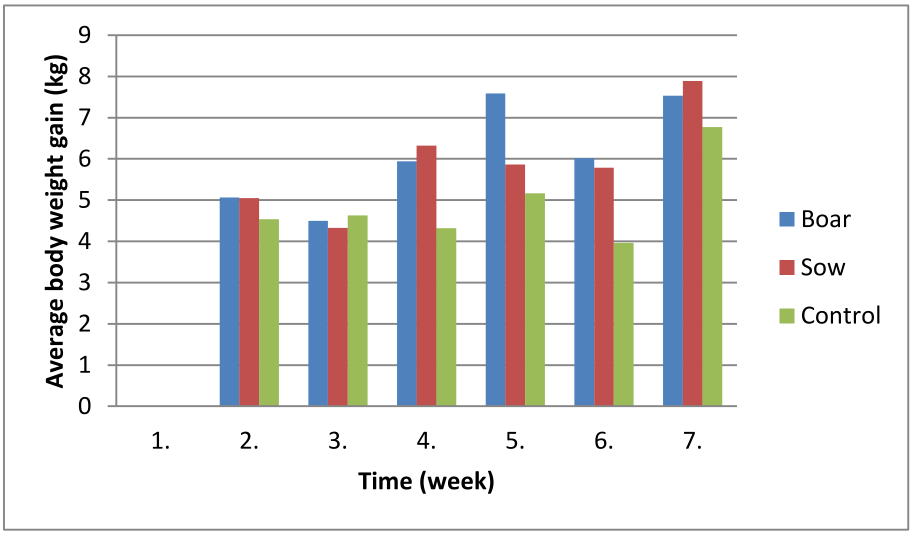

Table S2. The regression parameters of both linear models of BW growth versus time

Variante	Parametr			
	B_{2j}	B_{1j}	RSC_j	$s_{(e)}$
1. treated group	22.9267	6.1861	2437.0619	5.9866
2. control group	23.5850	4.9286	217.4945	2.3318
combination 1+2	23.1736	5.7146	3322.2524	5.4957

Note: Linear regression models $Y_{ij} = B_{2j} + B_{1j} * X_{ij} + \varepsilon_{ij}$ for M = 2 (models)

Table S3. Identification and confirmation of 17β-testosterone and testosterone esters by mass accuracy (MA) for MS[1], MS[2] data

Analyte	Elemental composition	Theor. precursor ion	Exp. precursor ion	Mass accuracy (ppm)	Element.com. Product ion	Theor. product ion	Exp. product ion	Mass accuracy (ppm)
17β-testosteron	$[C_{19}H_{28}O_2]^+$	289.21621	289.21609	-0.4	$[C_7H_9O_1]^+$	109.06479	109.06505	2.4
					$[C_6H_9O_1]^+$	97.06479	97.06491	1.2
testosterone propionate	$[C_{22}H_{32}O_3]^+$	345.24242	345.24207	-1.0	$[C_7H_9O_1]^+$	109.06479	109.06510	2,8
					$[C_6H_9O_1]^+$	97.06479	97.06519	2,9
testosterone isocaproate	$[C_{25}H_{28}O_3]^+$	387.28937	387.28864	-1.9	$[C_7H_9O_1]^+$	109.06479	109.06506	2,5
					$[C_6H_9O_1]^+$	97.06479	97.06520	2,2
testosterone decanoate	$[C_{29}H_{46}O_3]^+$	443.35197	443.35156	-0.9	$[C_7H_9O_1]^+$	109.06479	109.06506	2,5
					$[C_6H_9O_1]^+$	97.06479	97.06525	2,7
17β-testosteron-D2	$[C_{19}H_{26}O_2D_2]^+$	291.22876	291.22873	-0.1	$[C_7H_7O_1D_2]^+$	111.07700	111.07763	-1.1
					$[C_6H_7O_1D_2]^+$	99.07700	99.07780	-1.2

17β-testosterone-D2 is used as an isotopically labelled (D2) internal standard

Figure S2. The measured experimental MS[1] of the 17β-testosterone standards (up), comparison with the theoretical MS[1] (down)

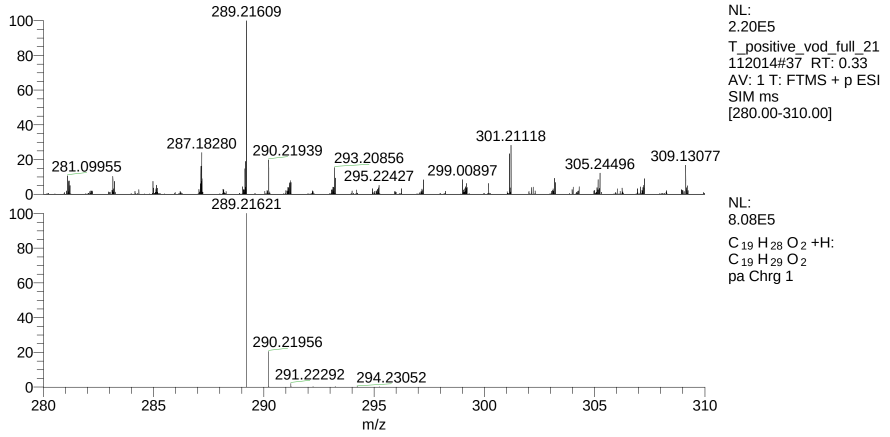

Figure S3. The measured experimental MS[1] of the 17β-testosterone-D2 standards (up), comparison with the theoretical MS[1] (down)

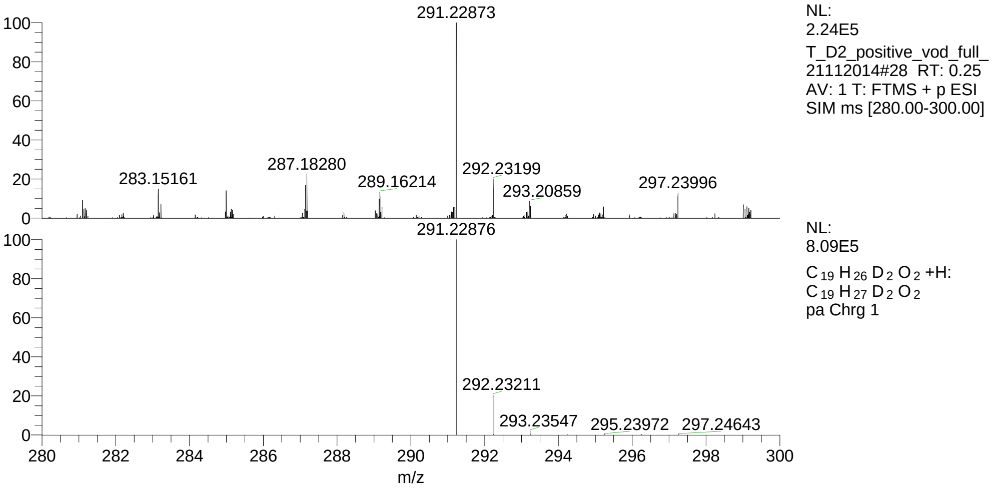

Table S4. Linearity of 17β-testosterone in plasma

Conc. [ng mL^{-1}]	Area Std.	Area IS-d2	Area Std/IS
80	24048803	4638666	5.184422202
80	26586189	5381880	4.939944592
80	27161633	5351732	5.075297679
80	27219150	5401907	5.038803889
40	14632261	5238167	2.793393376
40	14687974	5277390	2.783189039
40	13434587	4968167	2.704133537
40	13987231	5237554	2.670565497
20	7980994	5540146	1.440574671
20	10008477	6818326	1.467878919
20	8192988	5683542	1.441528540
20	9508455	6518765	1.458628283
10	4287842	5686560	0.754030908
10	4343003	5816020	0.746731098
10	4319507	5765321	0.749222290
10	4299832	5614298	0.765871708
5	2232106	5833132	0.382659950
5	2365280	5922302	0.399385239
5	2520885	6497156	0.387998226
5	3003206	7962756	0.377156603
2	1358004	6293905	0.215764934
2	1160846	5702955	0.203551668
2	1466863	6579850	0.222932590
2	1359639	6543118	0.207796803
0	182548	5979601	0.030528458
0	165905	5669014	0.029265230
0	184105	6384831	0.028834749
0	178111	6793122	0.026219314

Figure S4. Calibration curve of 17β-testosterone in plasma

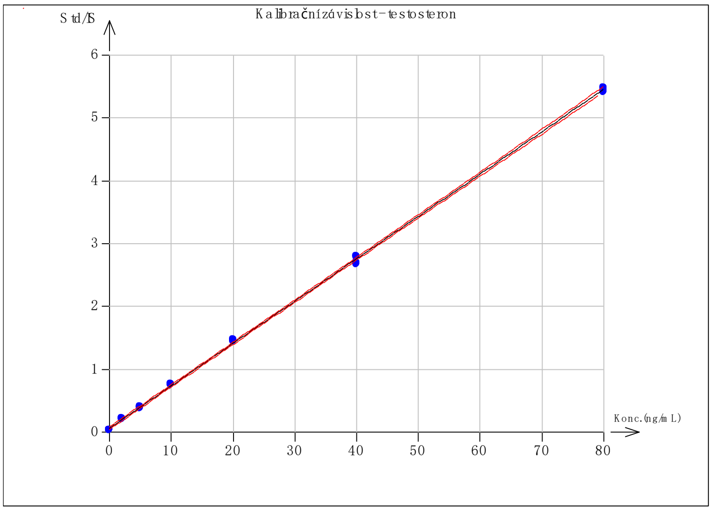

Table S5. Precision, repeatability and within-laboratory reproducibility

Iteration	Area 17**β**-testosterone / Area 17**β**-testosterone-D2<br>5 ng mL^{-1}	 10 ng mL^{-1}
1	0.3827	0.7540
2	0.3994	0.7467
3	0.3880	0.7492
4	0.3772	0.7659
5	0.3704	0.7461
6	0.3636	0.7639
7	0.3976	0.7620
8	0.3852	0.7538
9	0.3903	0.7434
10	0.3975	0.7569
11	0.3934	0.7396
12	0.4012	0.7512
Average	0.3872	0.7527
CI (95%) lower	0.3796	0.7475
CI (95%) upper	0.3948	0.7580
CV	0.000143	0.0000689
SD	0.0120	0.0083
CV (%)	3.09	1.10

Table S6. Pharmacokinetic of testosterone in plasma

	**Pig**	**Concentration 17β-testosterone (ng mL^{-1})**<br>**Day 0**	**Day 1**	**Day 2**	**Day 3**	**Day 7**	**Day 14**	**Day 21**
♂	Pig 1	2.02	31.40	23.41	8.44	1.55	3.25	2.02
♂	Pig 1	2.54	29.05	26.21	15.88	1.07	3.05	2.64
♂	Pig 2	1.03	22.52	15.19	15.35	5.32	3.48	1.08
♂	Pig 2	2.58	22.83	18.85	14.58	5.89	3.35	2.65
♂	Pig 4	1.56	36.25	24.42	17.14	4.73	4.96	1.58
♂	Pig 4	1.83	37.62	25.75	16.13	6.23	4.95	1.83
♂	Pig 5	1.38	33.53	22.13	18.27	7.26	1.66	1.27
♂	Pig 5	1.52	33.73	23.02	17.66	3.12	1.60	1.54
♂	Pig 7	0.61	23.63	21.24	13.12	5.92	3.04	0.58
♂	Pig 7	0.55	22.55	19.65	13.44	9.74	2.73	1.18
Average		1.56	29.31	21.99	15.00	5.08	3.21	1.64
SD		0.7089	6.0090	3.3826	2.8599	2.6197	1.1277	0.6681
♀	Pig 11	< LOD	44.32	34.07	24.19	9.88	4.90	0.22
♀	Pig 11	< LOD	40.17	30.91	19.27	10.46	0.82	0.29
♀	Pig 13	< LOD	29.03	22.43	14.16	9.41	3.34	0.54
♀	Pig 13	< LOD	28.64	22.43	13.90	8.73	3.36	0.49
♀	Pig 14	< LOD	20.70	15.54	6.11	7.27	3.39	0.02
♀	Pig 14	< LOD	23.19	17.35	16.03	7.21	3.22	0.03
♀	Pig 16	< LOD	35.21	19.69	9.30	3.92	2.41	0.11

♀	Pig 16	< LOD	32.59	20.44	11.67	6.86	2.37	< LOD
Average		< LOD	31.73	22.86	14.33	7.97	2.98	0.24
SD			8.0539	6.4442	5.6653	2.1098	1.1697	0.2110
				**control group**				
♂	Pig 8	0.79	0.36	0.50	0.29	0.83	0.30	0.80
♂	Pig 8	0.07	0.12	0.24	0.63	0.22	0.58	0.79
♂	Pig 9	0.67	0.25	0.32	0.16	0.55	0.27	0.74
♂	Pig 9	0.68	0.31	0.01	0.22	0.11	0.19	0.66
♀	Pig 12	< LOD	< LOD	< LOD	< LOD	< LOD	< LOD	< LOD
♀	Pig 12	< LOD	< LOD	< LOD	< LOD	< LOD	< LOD	< LOD
♀	Pig 15	< LOD	< LOD	< LOD	< LOD	< LOD	< LOD	< LOD
♀	Pig 15	< LOD	< LOD	< LOD	< LOD	< LOD	< LOD	< LOD
Average		0.55	0.26	0.27	0.32	0.43	0.34	0.75
SD			0.1016	0.2053	0.2119	0.3275	0.1694	0.0649

Note: LOD = 0.32 ng mL^{-1}

Figure S5. Hotelling plot for identification of outliers objects in data source matrix X with calculated critical value T2 for plasma, (K – control group vs. T – treated grup)

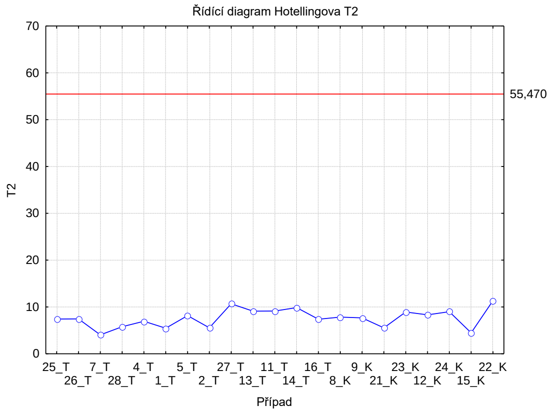

Figure S6. Hotelling plot for identification of outliers objects in data source matrix X with calculated critical value T2 for urine, (K – control group vs. T – treated group; M – male, F - female)

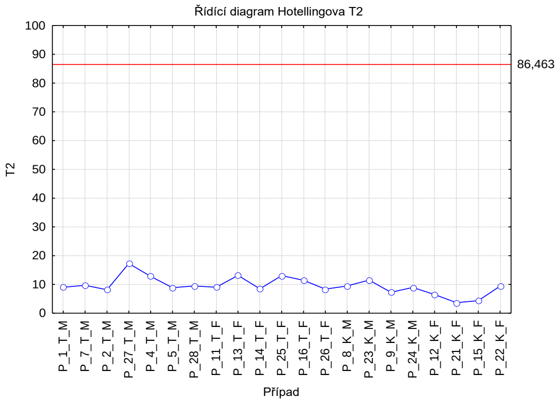

Figure S7. The PLS-DA score plots for plasma (A) and urine (B) data matrix demonstrates robust discrimination between the control group of pigs marked with blue color and the group of teated pigs marked with red color (Centering, Statistica).

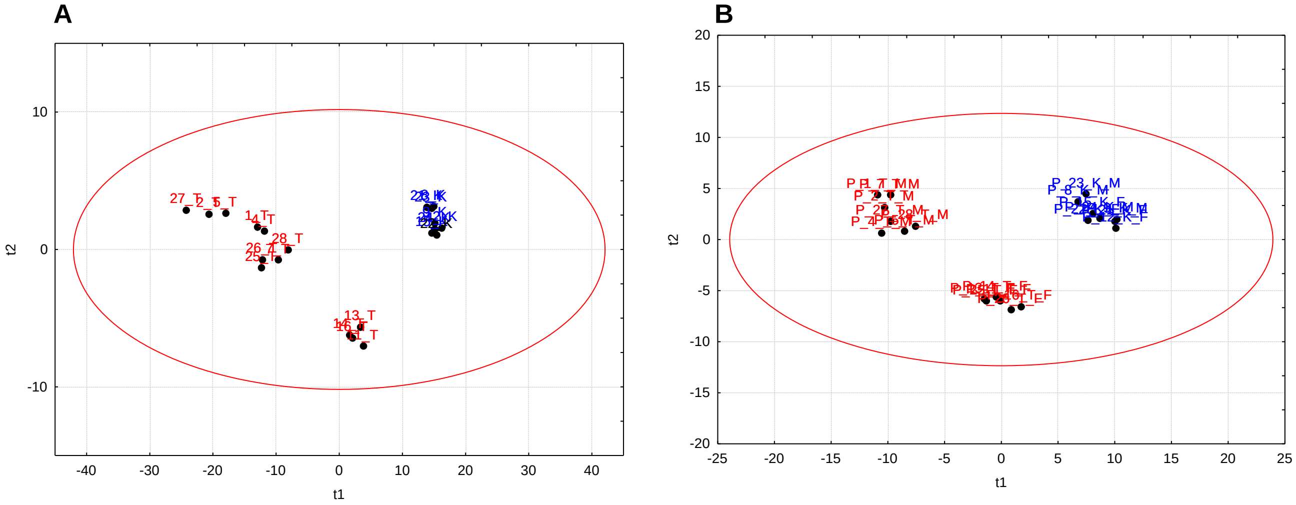

Figure S8. The OPLS-DA permutation tests further confirmed that the proposed statistical models are correct and robust; A – plasma, B – urine.

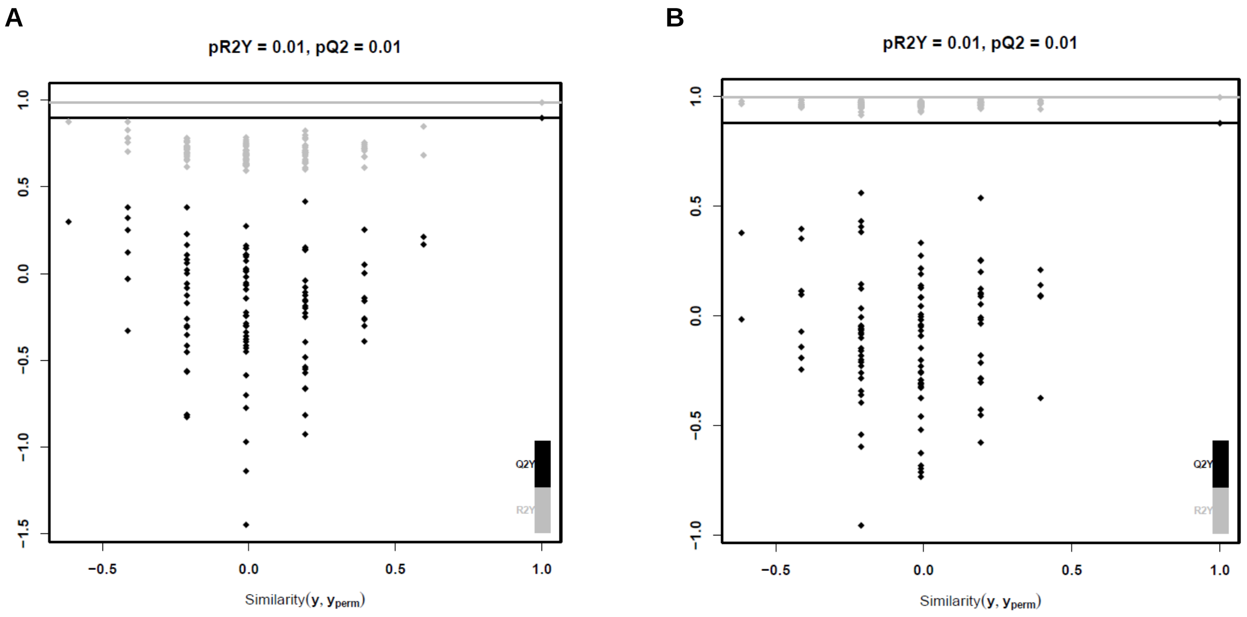

Figure S9. Variable importance in projection (VIP) and S-plots from OPLS-DA were used to determine the most discriminating metabolites between treatments and controls, A – plasma and B – urine.

A

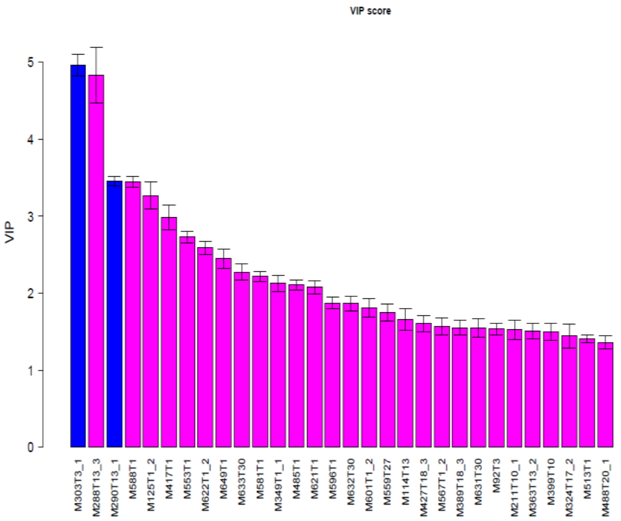

B

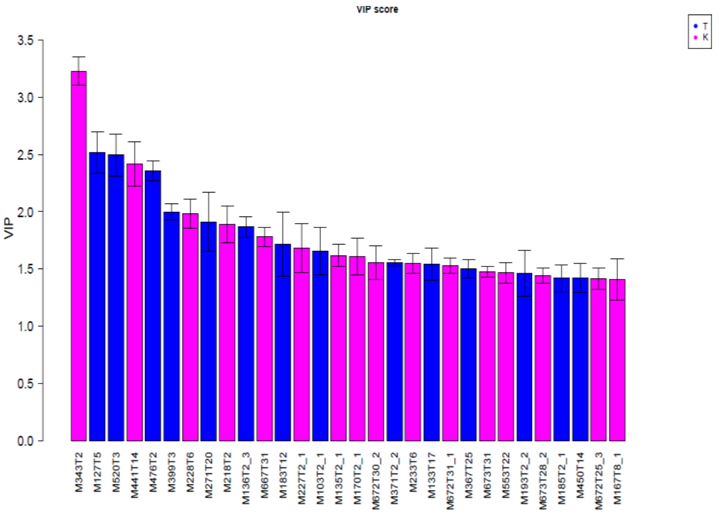

A

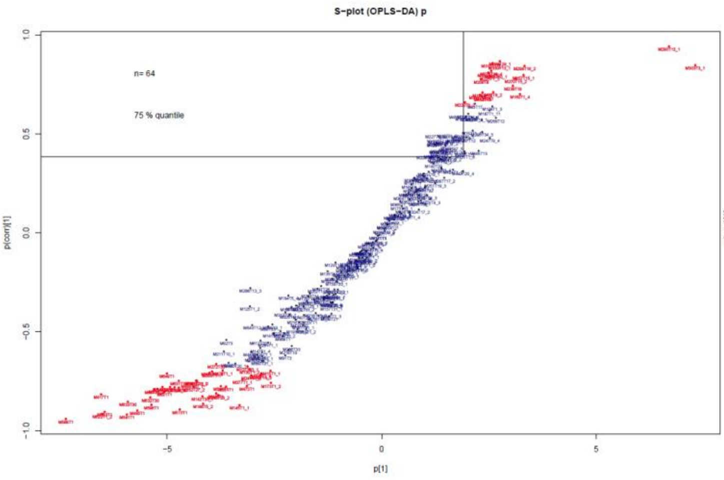

B

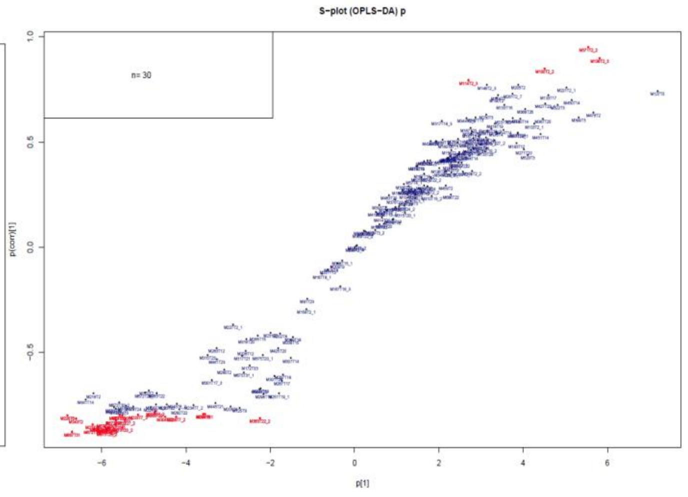

Supplement: Supplementary file 1 [file metabolites-10-00307-s001.pdf]
